# Early psychosis service user views on digital remote monitoring: a qualitative study

**DOI:** 10.1186/s12888-025-06859-4

**Published:** 2025-04-16

**Authors:** Sarah Trelfa, Natalie Berry, Xiaolong Zhang, Shôn Lewis, John Ainsworth, Katherine Berry, Dawn Edge, Gillian Haddock, Rohan Morris, Sandra Bucci

**Affiliations:** 1 Division of Psychology and Mental Health, School of Health Sciences, Faculty of Biology, Medicine and Health, Manchester Academic Health Science Centre, The University of Manchester, 2ndFloor, Zochonis Building, Brunswick Street, Manchester, M13 9PL UK; 2 Greater Manchester Mental Health NHS Foundation Trust, Manchester, UK

**Keywords:** Digital remote monitoring, Psychosis, Qualitative study, Framework analysis

## Abstract

**Background:**

Current approaches to mental healthcare for people with severe mental health problems are limited by sporadic monitoring and symptom recall bias. Emotional and behavioural markers generated by digital health technologies (DHTs) offer the potential to enhance quality of care and clinical decision-making. This study explored early psychosis service users’ views and experiences of using a digital remote monitoring tool (ClinTouch app).

**Methods:**

Qualitative framework analysis was undertaken with interview data collected from participants who took part in the Actissist proof-of-concept and subsequent randomised controlled trial studies to understand the experiences of participants using the ClinTouch app (*n* = 8).

**Results:**

Data were summarised into four key themes. The following three themes were established a priori: (1) awareness of mood and symptoms; (2) acceptability of ClinTouch; and (3) improvements and recommendations. The fourth theme was established a posteriori: (4) integrating ClinTouch into clinical practice. More specifically, participants felt ClinTouch was an acceptable and useful tool for symptom monitoring. ClinTouch facilitated an increased awareness of mood and symptoms, which enabled participants to self-reflect and develop understanding of their own experiences.

**Conclusions:**

This study shed light on early psychosis service users’ experiences with using the ClinTouch digital remote monitoring app. ClinTouch was viewed as acceptable for monitoring symptoms, safe and easy to use, showed potential of integration with clinical care, and facilitated increased awareness and understanding of symptoms. Improvements including personalised question items and interactive features were suggested. Future developments of digital remote monitoring apps should include a more refined item set and personalisation features.

**Clinical trial number:**

ISRCTN34966555, Registration Date: 12/06/2014; ISRCTN76986679, Registration Date: 07/02/2018.

**Supplementary Information:**

The online version contains supplementary material available at 10.1186/s12888-025-06859-4.

## Introduction

Psychosis is a severe mental health problem that affects an individual’s thoughts, feelings, perceptions and behaviours [1]. The typical age of onset of psychosis is during adolescence [2]. Psychosis results in disruptions to relationships, employment opportunities, educational performance, and health outcomes [3, 4]. Timely access to mental health services for treatment, such as that offered by early intervention for psychosis services (EIS), is critical to reducing or minimising its impact [5, 6], as evidence suggests that a long duration of untreated psychosis is associated with poorer personal recovery, increased service use and poorer economic outcomes [7, 8]. Current approaches to mental health assessment are often limited by sporadic monitoring from healthcare professionals, relying on patients’ retrospective symptom recall and requiring expertise to unpick the complex nature of information and experiences shared [9, 10]. This can result in barriers for implementing and accessing time-sensitive assessment and intervention [11, 12].

Digital health technologies (DHTs) could improve and/or scale up access to mental health support due to in part their widespread availability and ability to empower individuals in their clinical care [13, 14]. Emotional and behavioural markers generated by DHTs offer the potential to enhance quality of care and facilitate relapse detection [15]. Smartphones are the most advantageous digital tool for symptom monitoring given their ability to generate a plethora of data [16]. Active symptom monitoring (ASM) is one the common symptom monitoring methods using smartphone, typically defined as a user self-reporting data (e.g. psychological, behavioural, physiological) by ecological momentary assessment or other ambulatory assessment methods [17]. It usually requires the user to respond to a prompt and complete a set of questions or tasks multiple times on a smartphone app designed to track symptoms, functioning, or cognitive changes. Use of smartphone apps as self-monitoring tools offers a potential solution to overcome current challenges by assisting with assessment, collecting ecologically-valid symptom data, shaping treatment pathways and detecting relapse of long-term conditions [18, 19].

ClinTouch was one of the first apps to be used for ASM with people with severe mental health problems [20–22]. ClinTouch helps individuals experiencing severe mental health problems self-monitor their symptoms, with data from the app integrating into electronic health record systems to enable near real-time clinical monitoring [23, 24]. The ClinTouch items have been previously validated [21] through comparison with the Positive and Negative Syndrome Scale (PANSS) [25], with most items demonstrating moderate to high correlations between service user-reported and researcher-rated items. As such, it has been shown to be valid and feasible as a symptom assessment and monitoring tool for psychosis [21] and is particularly effective in bringing clinical benefit on psychotic symptoms for people accessing early intervention for psychosis services [23]. In the Actissist proof-of-concept trial and the subsequent powered RCT study (Actissist 2.0), ClinTouch was utilised as the active control condition to test whether Actissist, a cognitive behavioural therapy (CBT) informed digital intervention for psychosis, confers added benefit over and above symptom monitoring and to control for the non-specifics of using a smartphone. The proof-of-concept trial [26] found the Actissist app to be feasible, acceptable, and safe, with signals of improved psychotic symptoms in the Actissist group. However, the powered Actissist 2.0 RCT [27] showed that using both the Actissist app and ClinTouch app improved psychotic symptoms over time, but there was no differential effect between groups (i.e. using the Actissist app did not confer added benefit on psychotic symptoms at the primary end-point over and above using the ClinTouch app). At the end of the intervention period, we conducted exit interviews with participants randomised to both conditions in our overlapping Actissist [26] and Actissist 2.0 trials [27] and explored participants: i) experience of using the ClinTouch app for symptom monitoring; ii) views on the benefits of using the ClinTouch app for their mental health; and iii) challenges to using the app and recommendations for improvement. These are the findings we present in this paper.

## Methods

### Study design

This qualitative study was nested within the Actissist proof-of-concept [26] and powered efficacy [27] trials where we developed and tested whether the Actissist CBT-informed DHI, which targets relapse indicators of early psychosis, brought added benefit to help-seeking early psychosis service users over and above digital remote monitoring. In both trials, qualitative interviews were conducted with participants at the end of the trial exposure period (12 weeks in both trials) who received both the Actissist app (intervention arm) and ClinTouch app (active control arm). In this study, we report findings from qualitative exit interviews conducted with participants who received the ClinTouch app. Participant’s experiences of using the Actissist app are reported elsewhere (*in preparation*). We sought to understand participant’s experiences of using the app and further improvements that could be made. The study was reported in accordance with the Consolidated Criteria for Reporting Qualitative Research (COREQ) checklist [28] (See Supplementary Table 1).

### Recruitment and participants

Participants in both the Actissist proof-of-concept [26] and efficacy [27] trials were recruited from community mental health teams (CMHT) and early intervention for psychosis service (EIS) in the Northwest of England between March and September, 2015, and March 2018 and June 2020, respectively. Eligibility for taking part in both clinical trials were the same: (1) in current contact with CMHT or an EIS in the Northwest of England; (2) capacity to provide informed consent; and (3) English language proficient. Exclusion criteria were: (1) aged under 16 years at point of recruitment; (2) anyone with psychosis not in contact with an NHS mental health service; and (3) inpatient at point of recruitment. Participants who had taken part in the trials were eligible to be interviewed for the current study and were selected according to a sampling framework that focused on: participant gender, age, ethnicity, and app usage for the twelve-week study period (low, medium and high engagement). Of the 97 who were allocated to the ClinTouch condition in both trials, eight took part in the post intervention interviews; four from the Actissist proof-of-concept [26] and Actissist 2.0 [27] trials respectively. Ethical approval was obtained from the West of Scotland NHS Research Ethics Committee (REF: 17/WS/0221).

### ClinTouch app

**Fig. 1:**
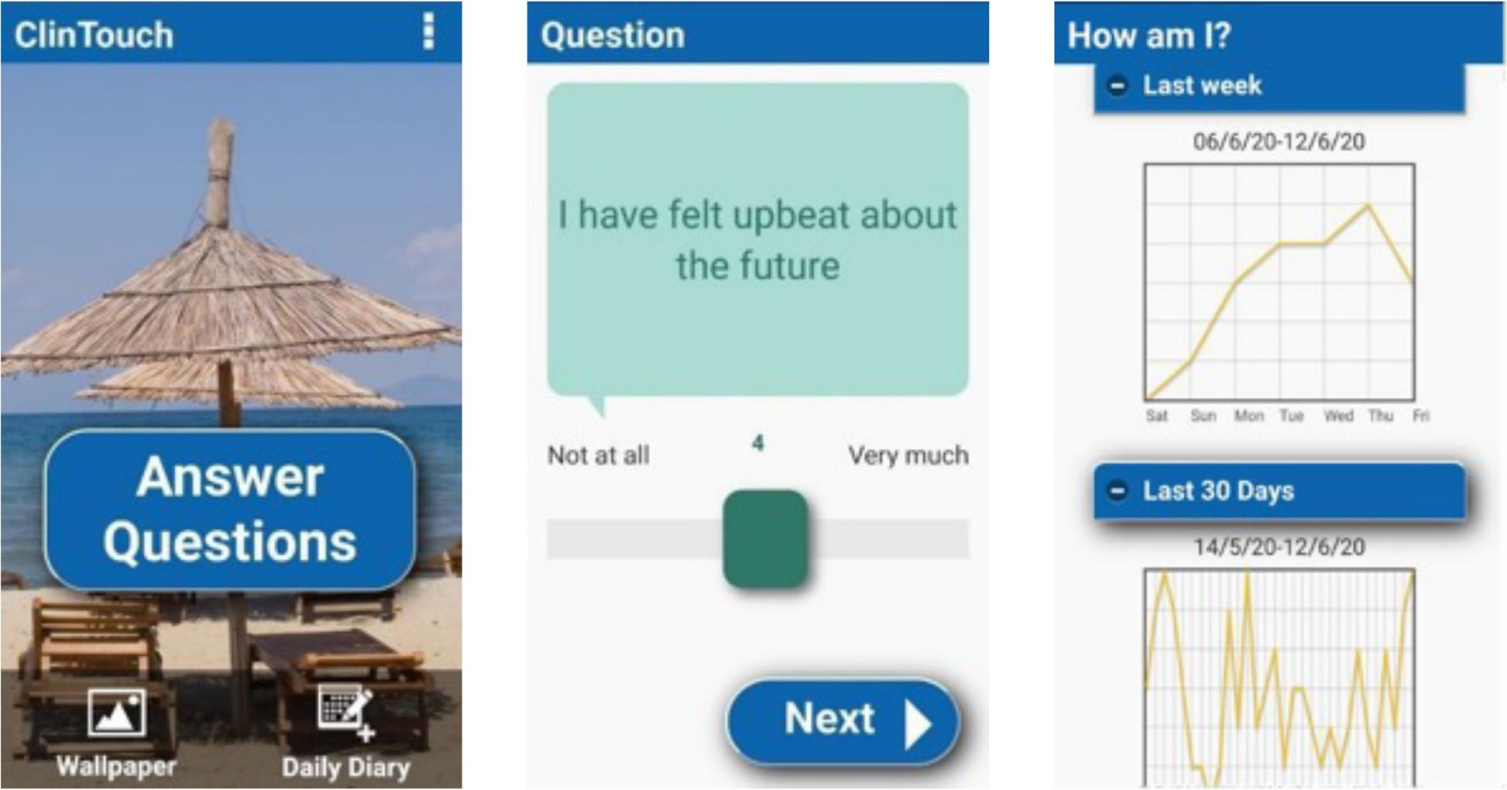
Screenshots of the ClinTouch app

ClinTouch [21] is a symptom-monitoring app that triggers, collects and wirelessly uploads symptom data to a server. Users are asked to record their symptoms at pseudo-random times throughout the day in response to an app notification. The notifications prompt users to answer a core set of items using a touchscreen slider to rate the severity of 12 individual mood, anxiety and psychotic symptoms on a 1–7 scale (see Table 1 for examples of the items). The items have been previously validated against the PANSS [25]. Summary symptom data and graphs showing change over time are available for users to view in the app. The notifications time out after 30 min, after which the notification is no longer visible, and the assessment items are no longer accessible. Screenshots of the ClinTouch app are shown in Fig. 1. In both Actissist trials [26, 27], the ClinTouch app was either installed on the participant’s own phone or a study smartphone, which was loaned to the participant for the duration of the intervention period. Participants were offered a 45-min onboarding session focused on basic use of the smartphone (eg, charging the phone; on/off), demonstration of the app, setting a passcode, navigating participants through the app domains and settings, and explaining the rationale for symptom monitoring. Written and visual “in-app” instructions were also available for participants to check. Participants had the opportunity to use the app and ask questions during the session. They were instructed to charge the phone regularly, to always carry the phone, and to go about their daily life as usual. No restrictions were placed on smartphone use for other purposes. Participants were reimbursed for data usage and for completing assessments and interviews with researchers, but not for engaging with the app itself.

**Table 1:** Examples of the ClinTouch items

Domain	Item	Format
Mood symptoms	‘I have felt sad’	7-point Likert scale
Anxiety symptoms	‘I have felt worried, nervous or anxious’	7-point Likert scale
Psychotic symptoms	‘I have heard voices’	7-point Likert scale

### Procedure

Consenting participants were invited for an interview following the 12-week intervention period. Prior to starting the interview, participants were asked to consent to the interviews being audio-recorded, reminded of their right to withdraw, and of the confidentiality process. Participants could choose whether to be interviewed at home or in a clinical setting and were reminded that taking part was optional and that interviews would explore their experience of using the ClinTouch app. A semi-structured interview topic guide (see Supplementary Table 2) was developed by the research team with input from a lived experience group and an expert reference group that comprised healthcare professionals, academics and individuals with lived experience of psychosis [29]. The topic guide was flexible in nature and refined throughout both trials and covered the following topics: (1) experiences and expectations of using the ClinTouch app; (2) views on the content, duration and intensity of ClinTouch; (3) engagement and integration of ClinTouch into everyday life and mental health care; (4) the utility of symptom-monitoring; and (5) recommendations for improvement. Questions were open-ended to elicit in-depth responses and often supplemented by additional prompts to facilitate elaboration of responses. The order of the questions asked were influenced by the topic guide but remained flexible and were not exclusively driven by the topic guide. Interviews remained iterative and inductive to any unanticipated themes, enabling the data to drive development of relevant questions. For example, as participants mentioned they were not aware of the graphs feature of the ClinTouch app, we added a question to ask about how the research team could make the graphs a clearer feature.

### Data analysis

Audio recordings were transcribed verbatim. NVivo version 12 [30] was used to analyse the data. Transcripts were independently coded by author ST under the supervision of authors NB and SB using a framework analysis approach [31], described in Table 2. Framework analysis is a systematic methodology that allows a combination of inductive and deductive coding. This approach was appropriate for the current study because it incorporates key themes already identified in the literature (a priori themes) and allows for unanticipated themes to be included (a posteriori themes). The topic guide and pre-existing literature were used to inform the framework’s a priori themes. We followed the proposed recursive and iterative six-stage analytical process to facilitate coding and theme-identification: (i) familiarisation with the data; (ii) generating initial codes; (iii) generating themes; (iv) reviewing potential themes; (v) defining and naming themes, and (vi) producing the study report [32]. Regular data analysis meetings were held between members of the research team to discuss and refine the analytical process, promoting methodological integrity. ST continuously familiarised themselves with the transcripts to become immersed with the data and used a reflective journal to reflect on the transcripts, which was used to aid interpretation of data.

**Table 2:** Description of the analytical process

Stage of Analysis	Description
1. Familiarisation	ST read all transcripts multiple times, making analytical notes
2. Coding the data	ST and NB each independently coded two interview transcripts. A priori codes were predefined by ST based on the topic guide, and inductive coding was used for other unanticipated, relevant topics
3. Developing the thematic framework	ST and NB met and discussed their coding with supervision from SB. Data were interpreted and summarised, new codes generated, and overlapping codes merged. A working analytical framework was developed
4. Indexing	ST recoded the two transcripts and applied the framework to the remaining six transcripts using QSR NVivo. Changes to the framework were discussed with SB and NB. A final framework was agreed, and ST recoded all transcripts
5. Charting	ST charted the data into a framework matrix for each theme and subtheme. Draft framework matrices were discussed by ST, NB and SB and necessary revisions were made
6. Mapping and interpretation	ST kept notes in a reflective journal during analysis. Mapped a priori and a posteriori characteristics of the data for interpretation

## Results

### Participant characteristics

Participant characteristics can be seen in Table 3. Participants were aged between 22 and 52 years (M = 30.6, SD = 9.9). Just over half were female (*n* = 5), unemployed (*n* = 6), and had a diagnosis of first episode psychosis (*n* = 5). Half were living with family (*n* = 4). Although all participants owned a smartphone (*n* = 8), some participants used a study phone if they preferred to install the app separate from their own smartphone or their own phone was not compatible with the app.

**Table 3:** Participant demographic data (*N* = 8)

Characteristic	Participants
**Age (years), mean (SD)**	30.6 (9.9)
**Gender, n (%)** ^a^	
Male	3 (38)
Female	5 (63)
**Ethnicity, n (%)** ^a^	
White British	6 (75)
White and Black Caribbean	1 (13)
Asian / Asian British—Bangladeshi	1 (13)
**Employment, n (%)**	
In education or training	2 (25)
Unemployed	6 (75)
**Highest level of education, n (%)** ^a^	
Did not complete secondary school	1 (13)
Secondary school	3 (38)
Further education	3 (38)
Higher education	1 (13)
**Living arrangements, n (%)** ^a^	
Private rental	2 (25)
Local housing authority	1 (13)
Homeowner	1 (13)
Living with family	4 (50)
**Diagnosis, n (%)** ^a^	
Psychotic depression	1 (13)
First episode psychosis	5 (63)
Acute and transient psychotic disorder	1 (13)
Bipolar disorder with psychotic features	1 (13)
**Actissist 1 or Actissist 2, n (%)**	
1	4 (50)
2	4 (50)
**Mobile phone ownership, n (%)**	
Yes	8 (100)

^a^Due to rounding, percentages may not add up exactly to 100%

**Fig. 2:**
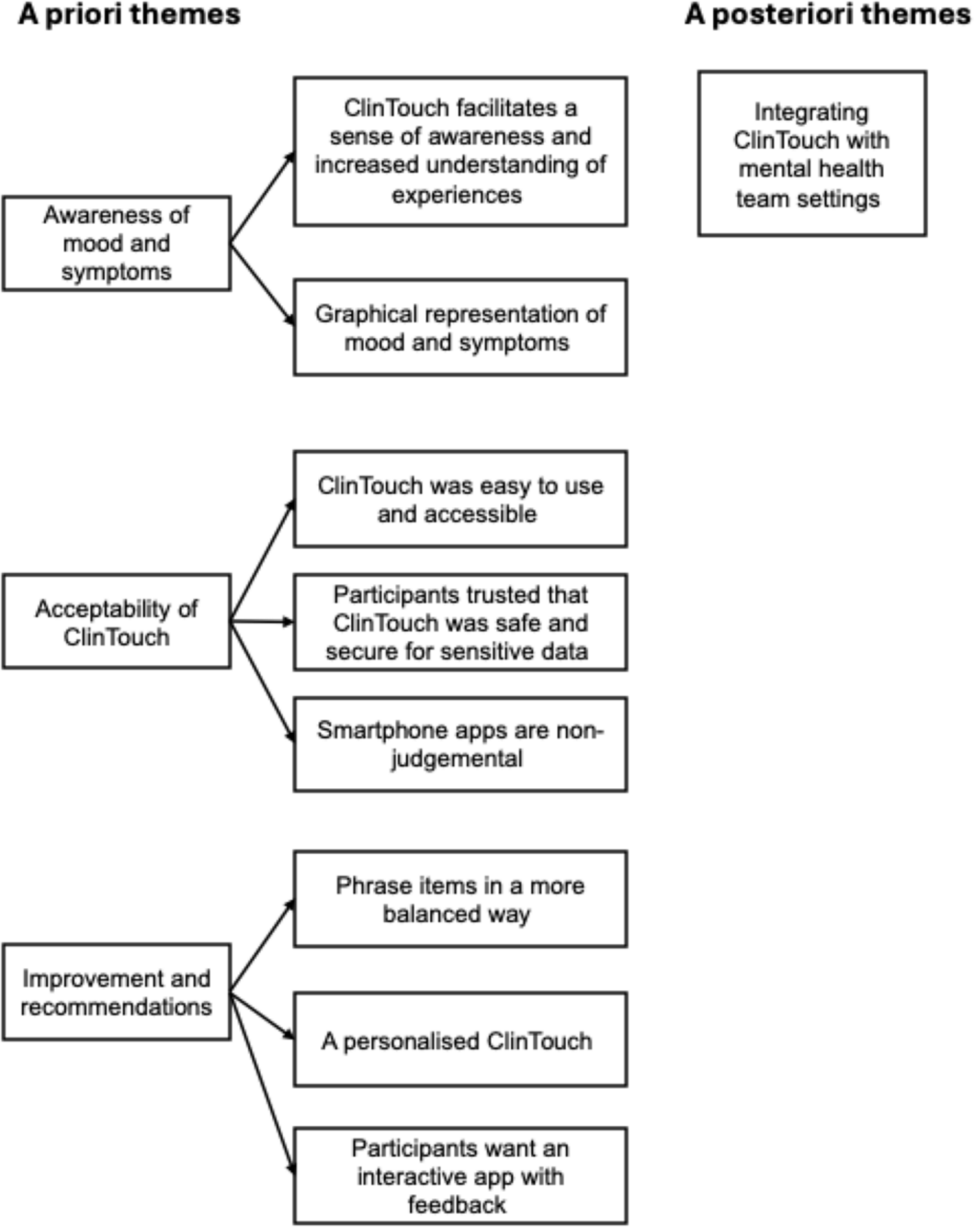
Summary of framework themes and subthemes

Three themes were established a priori: (1) awareness of mood and symptoms; (2) acceptability of ClinTouch; and (3) improvements and recommendations. One theme was generated a posteriori: (4) integrating ClinTouch with mental health team settings. An illustrative diagram of the framework is presented in Fig. 2 and described and elaborated below, as evidenced by key quotations embedded within the text.

### Theme 1: awareness of mood and symptoms

Participants described how they used ClinTouch to monitor their mood and symptoms. Specifically, answering questions about symptoms and using the graphs for visual representation of symptoms fostered a sense of understanding and awareness of symptom fluctuations and trends; although, for some participants, increased awareness of mood and symptoms was viewed as unhelpful.

#### ClinTouch facilitates a sense of awareness and increased understanding of experiences

The process of engaging with the ClinTouch app through answering questions multiple times throughout the week encouraged participants to connect with and self-reflect on experiences. This increased self-awareness helped participants to gain clarity of their subjective experiences and understand patterns of behaviour:

*It did make me think about my symptoms and when I seemed to experience them more or less … for example, when I am stressed, my symptoms increased and that was very noticeable when using the app.* [Participant 5]

Some participants used this self-awareness to seek and implement solutions, which facilitated self-management of symptoms:

*I’d look at the app and see what had happened within the week and I’d think well what can I do, what can I do to change that rather than me having a good week Monday to Wednesday and then it dipping in and then go back up again … it helps me manage my symptoms quite easily.* [Participant 3]

One participant felt that repeatedly recording their mood and symptoms brought negative thoughts and feelings to the forefront of their minds, which impacted on their use of the app:

*I was constantly reminded of like my symptoms … I felt like it brought me down a little and then I didn’t like want to carry on with the app and like follow it through.* [Participant 6]

Although reminders of mood and symptoms for this participant were viewed as unhelpful, their increased awareness facilitated the realisation that their experiences and difficulties were not improving. Consequently, the participant pursued change and independently sought alternative solutions, for example, psychological support:

*It did make me realise a bit more about like in terms of like how I was thinking and like that vicious cycle that I was putting myself in … I did like end up like more like calling and seeking therapy … that is something that I might have realised whilst using the app … I should be like also doing more.* [Participant 6]

#### Summary of mood and symptoms change

One perceived benefit of ClinTouch was the visual summary of mood and symptoms via the graphs generated by repeatedly recording symptom scores over time in the app. Participants were able to observe how their symptoms and mood changed over time, which afforded them insights into patterns and trends in their mood:

*With the chart as well, it made me realise my up and down and it was making me realise that I'm a bit more stable than I think I am … It actually showed me that well, I did a bit better but then I went up again and so it helped me like be able to stabilise my mood and control my mood a lot more.* [Participant 3]

The benefit of visual display of symptoms increased participant’s awareness of how their symptoms might be associated with specific situations or contexts. In turn, participants were able to implement strategies to reduce the reoccurrence of triggers in similar situations. In a similar vein, participants commented that the app facilitated documenting and tracking symptoms, translating subjective experiences into objective graphical data representing these experiences:

*It allows you to make like a mental note of how you’re feeling at that time, it lets you document what you are feeling. I did like the graphs because that could document my, like, you know, if I was having a really bad day, I could look back at it and see “oh but I did have a really good day”.* [Participant 5]

However, a few participants were unaware of the graphs on ClinTouch even though this was shown to them during the onboarding session, and felt they would have used this feature if they were aware of it. When asked how the research team could make the graphs a clearer feature, participants suggested: *tell me at the beginning to look at the graph”* [Participant 7] or have a notification *“pop up that said like here is the graph”* [Participant 1].

### Theme 2: acceptability of ClinTouch

ClinTouch was viewed as easy to use, accessible, safe and secure. However, there were mixed views expressed about using a smartphone app relating to their mental health for fear of judgement from others.

#### ClinTouch was easy to use and accessible

All participants found ClinTouch and its features simple to use and integrated the app with ease into daily life:

I think that it was quite straightforward and quite a lot of features were easy to use as well. [Participant 3]

I had one of your phones [the loaned study smartphone] and no, actually, dead easy to use … I think the slide thing is, yeah, it’s okay, like I said, it’s easy to use. There’s no doubt about that. [Participant 4]

Additionally, using ClinTouch was not time-consuming, and the fact that ClinTouch was downloaded on a smartphone made it accessible:

*It’s not really like disrupting anything, it’s just like playing a game or just sending a text to someone.* [Participant 2]

However, some participants felt that borrowing a study phone to use the app was a barrier to engagement. From this standpoint, participants felt the app would be better integrated into daily life if it was added to their personal smartphone:

*Not very well [fitted into my everyday life]… but that’s probably because obviously it was [in another phone] isn’t it, because obviously I couldn’t just download it on my phone or something, that probably would have been better, but that’s because my phone’s like a terrible phone or something … so that’s probably why I got the [study] phone.* [Participant 6]

#### Participants trusted that ClinTouch was safe and secure for sensitive data

Participants were asked about whether they felt ClinTouch was safe to use and secure; all participants felt that it was. Some participants noted that they trusted ClinTouch with their data because the app was *“coming like from a university”* [Participant 2]. Detailed explanations regarding data security and data protection at the start of the study aided participant confidence in the handling of their data:

*I felt quite secure because, I knew, I knew there it was quite high security and I was told at the beginning that it was all confidential.* [Participant 3]

Whilst many participants did not express concerns about the security and storage of their data, a few participants expressed they would not wish for their data to be accessed without their consent:

*I wouldn’t want anyone else to access it because I feel like that my symptoms are mine … that’s my information that I, only I should be able to access and nobody else.* [Participant 6]

#### Smartphone apps are non-judgemental

A couple of participants preferred using ClinTouch to answer questions compared with face-to-face settings and felt that the app had a *“clinical detachment”* [Participant 1] to answering questions related to mental health:

*Telling the app how I felt, felt like I wasn’t being judged because it wasn’t a person.* [Participant 2]

However, one participant described feeling embarrassed answering questions on ClinTouch while other people were around as they felt uncomfortable disclosing their mental health to others:

*I didn't want that to sort of... you know for them [other people around] to have been “ugh right, is that how she's feeling, are you hearing voices” or that kind of thing, I didn't want that, sort of that little bit embarrassed by them questions, I felt like I had to sort of hide it.* [Participant 4]

Another participant felt that the large font size and background colour of the questions made ClinTouch more noticeable compared to other apps, which made them feel perceptible to judgement from others: *“anybody can read the words on the screen”.* [Participant 1].

Overall, while some participants felt that smartphone apps minimise the risk of judgement, others perceived that using the app could make them more vulnerable to judgement from others.

### Theme 3: improvements and recommendations

Reflecting on the usefulness of ClinTouch, participants felt the app was limited by the phrasing of the questions and should encompass self-help solutions to enable self-management. Participants offered some suggestions to further develop the app, for instance, by including more personalisation features.

#### Phrase items in a more balanced way

Some participants mentioned negative reactivity (changes in thoughts or emotions) from answering the questions related to how the questions were worded/phrased. For example, the negative valence wording of questions led them to respond in a negative way:

*[The questions make me feel] worse I think, it is good to ask these questions but also it’s not as well, because for that second or for that little period, it’s making you feel like, like this one, “I feel like the future holds little for me.” Er, you know, urgh, oh no, does it? Well, no, it doesn't, so I'm going to feel more crap.* [Participant 4]

One participant reflected on their experience when answering the questions and queried whether the act of responding to questions itself changed their emotional state. To mitigate this, participants recommended phrasing questions in a more neutral or positive way to minimise how influenced the user felt by how the question was asked:

*Some of the questions they are kind of emotive things in them in themselves so you’re not necessarily really answering specifically how you feel because the apps changed how you feel.* [Participant 1]

#### A personalised ClinTouch

The items and contents of the ClinTouch app were viewed as generic by many participants, which was considered a limitation of the app as the way in which questions were asked in the app was not tailored to the end user’s individual circumstance:

*It wasn’t very personalised it was just very generic, generic questions and generic rating scales, and then it didn’t, at the end it just said “well done you’ve finished the questions”.* [Participant 5]

Moreover, participants sometimes found it difficult to relate their experiences to a selection of specific pre-determined questions. They suggested that questions could be personalised to individuals’ mental health difficulties:

*It could be more meaningful as in have I thought about my illness today, have I shown a particular symptom that particularly worries me or stresses me out or makes it more difficult just to go about my day.* [Participant 1]

Some participants felt ClinTouch was repetitive and monotonous, which subsequently effected their engagement and usage over the 12-week intervention period as they lost interest:

*The repetitiveness of it, you just keep answering the same questions, which I didn’t do towards the end. I’d lost interest, it was a bit long.* [Participant 8]

Most participants wanted the ability to personalise app alerts. The ability to answer questions at convenience was viewed as having the potential to improve engagement with the app and enable participants to answer the questions without fear of judgement from others:

I’d say with the scheduling of them, if I had some sort of control over that then probably be better at it. [Participant 1]

If it’s going off and you’re not in a convenient place at a convenient time … then it should give you when you want to come back to it kind of thing … so when you got that spare little bit of time, go back to it, because these people around you, you can’t just like … ‘cause then I don't feel like you're going to be answering the questions honestly … you just want to get through it quicker. [Participant 4]

One participant suggested an initial demo test of the app to personalise the alerts schedule as a solution to overcome missing alerts because of inconvenient scheduling of alert notifications:

*It might have also been helpful to have like a like a couple of days sort of initial demo test of it [ClinTouch] before any of that went on … like a day of just like running through, that would have been useful … then you could have got the times [the schedule of the alerts] right as well.* [Participant 1]

#### Participants want an interactive app with feedback

Participants felt that ClinTouch could be improved by including feedback in the form of suggested solutions and self-help resources in response to the questions asked. Without this, participants felt they were left alone thinking about their mood and symptoms without support to manage these difficult thoughts and feelings:

*It gives you no feedback, it’s like saying how do you feel? It’s like anybody asking you how you feel, then going “sweet” … like walking off.* [Participant 1]

Some participants also believed that their engagement with ClinTouch would have increased if it had more functions, such as links to external resources:

*If it’s like a news type thing and you’ve got this app on your phone and yeah it prompts you to do stuff, but it also is a thing you can click on and be like, well there was this article on it or some vital piece of information that was actually interesting at the same time as doing this stuff then can I have it for a lifetime? Useful.* [Participant 1]

Participants believed that symptom-monitoring alone was not enough to help self-manage their mental health and identified the need to incorporate interactive and feedback loops including self-help resources and solutions.

### Theme 4: integrating ClinTouch with mental health team settings

Several participants described how they helpfully used the data gathered in ClinTouch during appointments with members of their clinical team; for example, during care coordination sessions:

*I showed her [care coordinator] the graph because like it sometimes it peaked and then it came back down again and peaked again, then she was asking about the two things that I was doing … she was trying to give me, you know, some things around it.* [Participant 2]

The increased awareness of mood and symptoms together with the facility to record experiences in real-time provided participants with an opportunity to collaborate with the mental health team and inform treatment decisions in a way that they had not previously been able to achieve. In this sense, ClinTouch users felt empowered to take more agency and control of their mental health journey and interactions with their care team through more shared decision-making (between the participant and clinical team):

*Say that you were speaking to someone like a psychiatrist, and you can say “well I’ve been through on my app, and this is what it’s showing right now” and they would be able to help alongside … rather than saying to someone “right take this it’s going to do this for you”.* [Participant 3]

One participant speculated whether having access to the symptom-monitoring app at an earlier point in their mental health journey would have facilitated a shared understanding of their experiences with the mental health team and influenced the treatment they received at an earlier point on their journey:

*The app really helped and I said that and so my doctors finally put my forward to CBT but this [psychosis] is three years on…maybe if that would be a solution before … say if I always had the app then I do think that would make a real difference.* [Participant 3]

Overall, participants saw value of and utility in integrating the data gathered in the ClinTouch app with their clinical team, especially the ability to show and evidence their mood and symptom experiences recorded in real-time with team members. Some participants also suggested that using the app at an early stage of mental health care may have brought greater benefits to their overall mental health journey.

## Discussion

Building on previous studies on ClinTouch [21, 23, 24], this study further demonstrated that ClinTouch was an acceptable and useful tool for symptom monitoring for early psychosis service users. More specifically, we found that ClinTouch facilitated an increased awareness of mood and symptoms, which enabled participants to self-reflect and develop understanding of their own experience. While the questions negatively impacted on mood and symptoms for some participants, the negative reminders of an unchanging situation did facilitate help-seeking via non-digital routes. Data visualisations through graphs of symptom fluctuations over time afforded insights into symptom trends and patterns, which facilitated self-awareness and self-management. This enabled some participants to self-manage their mental health through implementing strategies to respond to observed symptom patterns, which may explain its effects on improving psychotic symptoms. However, some participants were not aware of the graph feature and suggested specific reminder prompts would be beneficial for accessing this feature. These findings closely align with the broader literature which suggests that digital remote monitoring is viewed as an effective tool to aid understanding of psychosis through self-reflection and gain new insight on their experiences over time [33–35].

Participants viewed ClinTouch as a platform to develop awareness of mental health and voice their choice towards decisions. Some participants felt that ClinTouch reduced a perceived sense of judgement as assessment questions came from a faceless device, rather than a person. Additionally, some participants actively used ClinTouch with mental healthcare staff to aid communication about their experiences and show symptom and mood fluctuations. As such, this empowered some participants to engage more actively with their mental health team and participate in shared decision-making, highlighting digital monitoring as a potential tool to bridge the gap in collaborative treatment decisions between mental healthcare staff and individuals accessing psychosis services [36, 37].

The fact that ClinTouch came from a trusted source, in this case an academic institution, contributed to its acceptability. Berry et al. [38] echo these findings, where service users reported they would only feel comfortable answering questions if the app was from a trusted source, such as a university or the NHS. Whilst previous studies have found that service users expressed concerns about sharing data from a DHT with their mental health team, mainly due to worries about third party access to their data, safe data storage with mental health teams, and the impact on their therapeutic relationship [38, 39], we found that participants were open to the idea of automatically sharing data with their mental health team if detailed explanations regarding data security and data protection were provided. These findings may reflect the differences between hypothetical acceptability and actual acceptability [40], where participants in this study experienced using a DHT such that they may have felt more comfortable in the knowledge of what exactly is being shared with their mental health team. This suggests that offering service users more opportunities to engage with DHTs may help alleviate concerns and improve their acceptability with clinical integration.

Participants reported several areas of improvement for the app. As suggested by de Thurah et al. [41], incorporating lived experiences of people with psychosis is essential to ensuring digital remote monitoring tools align with user needs. In their qualitative study with nine individuals with lived experience of psychosis regarding the use of Experience Sampling Methods (ESM) in clinical management, de Thurah and colleagues found that participants expressed a desire to monitor a diverse range of daily-life experiences and emphasised the importance of personalisation in ESM tools to enhance clinical utility. While recognising the potential of ESM to increase self-awareness and control over their mental health, some participants voiced concerns about the validity of self-monitoring and the potential burden it may impose. We highlight here the improvements participants suggested and discuss what future iterations should incorporate to integrate the needs of individuals with lived experience. The repetitive nature of questions was viewed as an unhelpful reminder of symptomatic status and negative mood, with some participants becoming preoccupied with past negative experiences. This is consistent with previous findings where repeated symptom and mood assessment was an unwelcome reminder of negative mood that users would rather forget [42]. Future iterations should explore integrating features on symptom-monitoring apps to promote positive feelings and help users manage negative thoughts elicited through symptom-monitoring. In line with other digital health studies [35, 43], participants said that the lack of feedback, interactive features or a supportive or helpful responses from the app negatively impacted their motivation for continued engagement with ClinTouch, limiting its utility over time. Including self-help resources in future iterations of the app would overcome this limitation.

### Strengths and limitations

Qualitative interviews helped to capture participants’ views on using a symptom-monitoring app for psychosis. Methodological rigour in this study ensured both the credibility and reliability of findings through regular consensus meetings with the wider research team to obtain feedback on data analysis and reflexivity [44]. Reflective journals were kept by the research team throughout data collection and analysis, which facilitated researcher awareness and acknowledgement of the influence of researcher beliefs on interpretation [45]. There are also some limitations. Time and resource constraints meant that the researcher was unable to contact participants for member checks to provide feedback on the framework and findings. Data analysis did not involve an individual with lived experience of psychosis due to time and financial constraints. The involvement of individuals with lived experience throughout the research process can offer valuable insight on complex findings that would not have otherwise been established [46]. Due to the voluntary nature of participation in the exit interviews, as well as practical constraints such as participant availability and willingness to engage after the trial, the number of participants who agreed to take part in an exit interview was limited. It is possible that those who chose to participate in this study had particularly positive or negative experiences with the ClinTouch app, which may have influenced the findings. Therefore, the views and experiences expressed are only reflective of a limited number of ClinTouch users. We acknowledge that repeated self-reporting of symptoms may introduce suggestibility effects, where simply asking certain questions could influence symptom perception and endorsement; future research should explore this possibility in more detail.

## Conclusion

This study demonstrates that ClinTouch was viewed as acceptable for digital remote monitoring, safe and easy to use, showed the potential of integration with mental healthcare, and facilitated increased awareness and understanding of symptom trends. Several improvements were suggested, such as providing personalised feedback from the app to improve engagement and clinical utility of the app. These findings have important implications in improving the design of future digital remote monitoring tools by including personalisation features, meaningful feedback and interaction, and accessible graphical representations of mood and symptoms.

## Supplementary Information

Supplementary Material 1.

## Data Availability

The data sets generated during or analysed during this study are available from the corresponding author on reasonable request.

## Abbreviations

ASM: Active symptom monitoring
CBT: Cognitive behavioural therapy
CMHT: Community mental health team
COREQ: Consolidated criteria for reporting qualitative research
DHT: Digital health technology
EIS: Early intervention for psychosis service
PANSS: Positive and Negative Syndrome Scale
RCT: Randomised controlled trial
