# Supplementary material for: Early psychosis service user views on digital remote monitoring: a qualitative study
Source: BMC Psychiatry. 2025 Apr 16;25:386. doi: 10.1186/s12888-025-06859-4 (PMC12004715; doi:10.1186/s12888-025-06859-4)
Supplement: Supplementary file 1 — Supplementary Material 1. [file 12888_2025_6859_MOESM1_ESM.docx]

#### **Supplementary Table 1. Topic Guide Used in Qualitative Interviews**


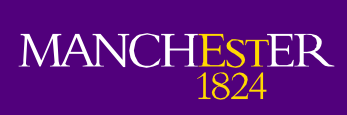

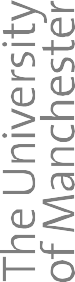


Qualitative Interviews (ClinTouch Arm)

Topic Guide

| **Participant Number** |  |  |  |  |  |
| --- | --- | --- | --- | --- | --- |

**Notes can be made on this topic guide; however, please ensure that no identifiable information is included in these notes. At the end of the topic guide there is a reflective log to complete. Please ensure that this log is completed after each participant and that actions based on the log are taken to the project management group as soon as possible.**

**Contents:**

**General interview guide and introductions Topic guide**

**Reflective log**

| **Prior to interview:** | Project officer will inform relevant research assistant based on sampling strategy to protect the blind.  Book in available timeslot |
| --- | --- |

# General Interview Guide

| **Equipment required:** | Participant Information Sheet (in case participant wants to see sheet again) Consent form (in case participant wants to see consent form again) Encrypted audio-recorder  Demo app version on a mobile phone |
| --- | --- |

**Introduction to Interview**

| **Introduction:** | Introduce self, welcome, and thank participant for attending the interview. Ensure that the participant is comfortable |
| --- | --- |
| C**onsent:** | Re-confirm informed consent is still valid and participant still wishes to take part. |
| **Interview details:** | Outline interview procedures and expected length of time for the interview.  Remind participant that the interview will be audio-recorded, that participation is voluntary they have the right to withdraw at any point without penalty and offer pauses and breaks. |
| **Explain the purpose of the interview and take any** | Thanks again for meeting with me today. In this interview, we will discuss your experience of using the ClinTouch app. The interview will take around  45 minutes to complete but may take a little bit more time or less time |

| **questions:** | depending on how much information you share. With your permission, the interview will be audio-recorded and then typed up to make sure we have an accurate summary of what you have said. Recordings will be securely stored in password protected computer files. We might use quotes that you provide during the interview in publications, but we would always make sure that you would not be identified in these quotes. Do you have any questions at all? |
| --- | --- |

## Topic Guide

| **What follows is a guide:** The order and exact content of the questions will be determined by the participant and will be influenced by the ongoing analysis so the order of the questions may vary as the interview develops.  *Probe and ask for examples as the time permits.* | |
| --- | --- |
| **Introduction to using the mobile phone app (ClinTouch):** | “Please could you describe what it was like using the ClinTouch app?”  ***Prompt:*** *were there any things about the app that you found particularly helpful?*  ***Prompt:*** *were there any things about the app that you didn’t find so helpful”*  “How did you feel about the app when you first saw it (i.e. at the beginning of the  project)?”  ***Prompt:*** *when you first saw the app was there anything you particularly liked?*  ***Prompt:*** *when you first saw the app was there anything you particularly didn’t like*  *or felt unsure about?*  “Have your feelings about the ClinTouch app changed since your first saw the app?”  ***if yes:***  ***Probe:*** *what has changed?*  “What have you learned from using the app?” |

|  | “Has using ClinTouch changed your daily life or behaviour in any way?”  ***If yes:***  ***Probe:*** *what has changed?*  ***Probe:*** *how did using ClinTouch help with this change?*  ***if no:***  ***Probe:*** *was there anything relating to your daily life or behaviour that you had hoped ClinTouch might have been able to help with?*  ***Probe:*** *why do you think ClinTouch didn’t help you with this?*  “Has using ClinTouch changed your thoughts and feelings about yourself in any  way?”  ***Probe:*** *what has changed?*  ***Probe:*** *how did using ClinTouch help with this change?*  ***if no:***  ***Probe:*** *was there anything relating to thoughts and feelings about yourself that you had hoped ClinTouch might have been able to help with?*  ***Probe:*** *why do you think ClinTouch didn’t help you with this?*  “Has using ClinTouch changed your views or understanding about psychosis in any way?”  ***Probe:*** *what has changed?*  ***Probe:*** *how did using ClinTouch help with this change?*  ***if no:***  ***Probe:*** *was there anything relating to your views or understanding about that you had hoped ClinTouch might have been able to help with?*  ***Probe:*** *why do you think ClinTouch didn’t help you with this?*  “Do you think ClinTouch has helped you?”  ***If yes:*** *How?*  “When we first gave you ClinTouch, we told you that the app is designed to help people track their experiences and symptoms over time. |
| --- | --- |

|  | “Do you think the app helped you with that?”  ***If yes or no:***  ***Probe:*** *why do you think this?*  ***Probe:*** *how does/does it not meet this purpose?*  *Probe: What could we have included to help us meet our intended purpose? What suggestions do you have to make it better?*  “Do you think this is a good way to manage symptoms for people with early psychosis?”  ***If yes or no:***  ***Probe:*** *why do you think this?*  “Has using the app made any changes to the way you manage your mental health?”  ***If yes:***  *Probe: please could you tell me more about the changes you have made and why?*  “Do you think you are aware of your mood and symptoms more now than before  using the phone?”  ***If yes:***  ***Probe:*** *Is this a good or a bad thing? Explore…*  ***Probe:*** *could you tell me more about how you are more aware than before?*  “How did you feel when it came to the end of the 12-weeks using the app?”  ***Prompt:*** *did you miss ClinTouch when you no longer had access to it?*  ***If yes:*** *in what way did you miss it?*  ***If no:*** *is there any reason why you didn’t miss having access to the app?*  “Would you want to use the app for longer than 12 weeks?”  *If so, why? If no, why not? What is the maximum length of time you think you would want to use it for?* |
| --- | --- |
| **Completing the questions and using the Multi-Media** | “You were given ClinTouch to use over a 12-week period. Did you make any changes to how you responded to the app during the time period?”  ***Probe:*** *could you tell me more about any changes in responding that you made?* |

| **content (MMM):** | “Do you feel you had enough information to help you to use the app?”  ***If yes:***  ***Probe:*** *what kind of information have you valued the most?*  ***Probe:*** *what kind of information have you thought was least helpful?*  ***If no:***  ***Probe:*** *what kind of information would you have found helpful and why?*  “Which questions were most relevant?”  ***If mention of specific questions:***  ***Probe:*** *why was this aspect of the app relevant?*  Was there anything missing in the app that you think we should have included?  ***Elaborate…***  “Were any questions more difficult than others?”  ***If yes:***  ***Probe:*** *could you tell me which ones/what made them difficult?*  ***If no:***  ***Probe:*** *were any questions easier to answer than others?*  “Are there any questions you did not want to answer?”  ***If yes:***  ***Probe:*** *can you give any reasons why?*  “What did you think about the number of questions asked and how often they  came?”  ***Probe:*** did your views on the number and how often *change over time?* |
| --- | --- |

|  | “What, if anything, could we have done to make it easier for you to answer the questions?”  *“*How could we make each question as meaningful for you as possible?”  ***Probe:*** *did you find any questions particularly meaningful/not meaningful?*  “Were the graphs helpful?”  ***Probe:*** *How/Why? How could we present the information in the graphs to make them more user friendly?* |
| --- | --- |
| **Fitting in with everyday life:** | “How well did using the phone/app fit into your everyday life?”  ***Probe:*** *Has it changed anything that you usually do?*  “Were there any times when the beeps interrupted what you were doing?  ***If yes:***  ***Probe:*** *what was it that you were doing?*  “Have you shown it to anyone else or discussed it with anyone else? What were their views?”  ***Prompt:*** *Did you show or discuss ClinTouch with your care coordinator or psychiatrist?*  ***if yes:*** *was this helpful at all? What was their response like? Did this impact or change the care that you received, if yes, in what way?*  ***if no:*** *was there any particular reason why you didn’t discuss ClinTouch with your care coordinator or psychiatrist?*  ***Prompt:*** *Did you show or discuss ClinTouch with any family members or friends?*  ***if yes:*** *was this helpful at all? What was their response like? Did this impact or change the care that you received, if yes, in what way?*  ***if no:*** *was there any particular reason why you didn’t discuss ClinTouch with family*  *members or friends?*  “If you were prompted to complete the questions whilst with other people, did you tell them about it?”  ***If no:*** |

|  | ***Probe:*** *what did you say instead?*  “Did you feel that using the phone ever felt part of your normal routine?”  ***Probe:*** *could you tell me more about why it did/didn’t?*  “How long do you think you could or would use the app for? Do you think 12 weeks is too long / not long enough?”  ***Probe:*** *what would be an ideal time for you?* |
| --- | --- |
| **What could be improved:** | “How could we improve the ClinTouch app?”  ***Probe:*** *can you think of anything in terms of the way the app worked or the content of the app?*  “Privacy and Safety - did you feel that the app was safe? Did you have any privacy concerns while using the app?”  ***If yes:***  ***Probe:*** *what were you concerned about?*  ***Probe:*** *why did this concern you?*  “Did you have any difficulties using the phone or the app at the beginning of the study?”  ***Probe:*** *this could be a technical problem or something to do with content?* |
| **Any other information about ClinTouch not already covered** | “We’ve discussed the app in quite a lot of detail now, before we talk about your experiences of taking part in this project more generally, was there anything else you’d like to tell me specifically about your experiences of using the app? |
| **Benefits and problems:** | “Now just thinking about the project more generally…” “Were there any benefits to taking part in the project?” ***Probe:*** *could you tell me why they have been a benefit?*  “Were there any negative consequences for taking part in the study?”  ***Probe:*** *could you tell me why that has been a difficulty?* |

|  | “Is there anything you would change about the process?”  ***If yes***  ***Probe:*** *could you tell me more about that?*  ***Probe:*** *why would you change this?* |
| --- | --- |
| **Interview Closedown:** | “Is there anything else that you would like to tell me that we haven’t discussed, but  you think might be relevant when thinking about the ClinTouch app or project?” “How have you found this interview today?”  “We’ll be interviewing quite a lot of people about their experiences of being involved in the project, are there any other questions that you think might have been helpful to ask?”  “Ok I’ll now switch of the audio recorder.” |
| **End of Interview:** | Explain what will happen with the information provided.  Ask whether it would be ok to contact the participant in a few months to double check they agree with the interpretation of their information.  Ask the participant whether they would like to receive a summary of the results.  Ask the participant whether they have any questions. |

#### **Supplementary Table 2. Consolidated Criteria for Reporting Qualitative Research (COREQ) Checklist**

Consolidated criteria for reporting qualitative studies (COREQ): 32-item checklist

Developed from:

Tong A, Sainsbury P, Craig J. Consolidated criteria for reporting qualitative research (COREQ): a 32- item checklist for interviews and focus groups. *International Journal for Quality in Health Care*. 2007. Volume 19, Number 6: pp. 349 – 357.

| **No. Item** | **Guide questions/description** | **Reported on Page #** |
| --- | --- | --- |
| **Domain 1: Research team and reflexivity** | | |
| *Personal Characteristics* |  |  |
| 1. Inter viewer/facilitator | Which author/s conducted the interview or focus group? | Methods |
| 2. Credentials | What were the researcher’s credentials?  E.g. PhD, MD | Methods |
| 3. Occupation | What was their occupation at the time of the study? | Methods |
| 4. Gender | Was the researcher male or female? | N/A |
| 5. Experience and training | What experience or training did the researcher have? |  |
| *Relationship with participants* | | |
| 6. Relationship established | Was a relationship established prior to study commencement? | N/A |
| 7. Participant knowledge of the interviewer | What did the participants know about the researcher? e.g. personal goals, reasons for doing the research | N/A |
| 8. Interviewer characteristics | What characteristics were reported about the inter viewer/facilitator? e.g. Bias, assumptions, reasons and interests in the research topic | Methods |
| **Domain 2: study design** |  |  |
| *Theoretical framework* |  |  |
| 9. Methodological orientation and Theory | What methodological orientation was stated to underpin the study? e.g. grounded theory, discourse analysis, ethnography, phenomenology, content  analysis | Methods |
| *Participant selection* |  |  |
| 10. Sampling | How were participants selected? e.g. purposive, convenience, consecutive, snowball | Methods |
| 11. Method of approach | How were participants approached? e.g. face-to-face, telephone, mail, email | Methods |
| 12. Sample size | How many participants were in the study? | Methods |

| 13. Non-participation | How many people refused to participate or dropped out? Reasons? | N/A |
| --- | --- | --- |
| *Setting* |  |  |
| 14. Setting of data collection | Where was the data collected? e.g. home, clinic, workplace | Methods |
| 15. Presence of non- participants | Was anyone else present besides the participants and researchers? | N/A |
| 16. Description of sample | What are the important characteristics of  the sample? e.g. demographic data, date | Results |
| *Data collection* |  |  |
| 17. Interview guide | Were questions, prompts, guides provided by the authors? Was it pilot  tested? | Methods |
| 18. Repeat interviews | Were repeat inter views carried out? If yes,  how many? | N/A |
| 19. Audio/visual recording | Did the research use audio or visual recording to collect the data? | Methods |
| 20. Field notes | Were field notes made during and/or after the inter view or focus group? | N/A |
| 21. Duration | What was the duration of the inter views or focus group? | N/A |
| 22. Data saturation | Was data saturation discussed? | N/A |
| 23. Transcripts returned | Were transcripts returned to participants for comment and/or correction? | N/A |
| *Data analysis* |  |  |
| 24. Number of data coders | How many data coders coded the data? | Methods |
| 25. Description of the coding tree | Did authors provide a description of the coding tree? | Results |
| 26. Derivation of themes | Were themes identified in advance or derived from the data? | Methods |
| 27. Software | What software, if applicable, was used to manage the data? | NVivo |
| 28. Participant checking | Did participants provide feedback on the findings? | Strengths and limitations |
| *Reporting* |  |  |
| 29. Quotations presented | Were participant quotations presented to illustrate the themes/findings? Was each quotation identified? e.g. participant  number | Results |
| 30. Data and findings consistent | Was there consistency between the data presented and the findings? | Results |
| 31. Clarity of major themes | Were major themes clearly presented in the findings? | Results |
| 32. Clarity of minor themes | Is there a description of diverse cases or discussion of minor themes? | Discussion |
